# RNA interference-mediated knockdown of 3, 4-dihydroxyphenylacetaldehyde synthase affects larval development and adult survival in the mosquito *Aedes aegypti*

**DOI:** 10.1186/s13071-019-3568-7

**Published:** 2019-06-24

**Authors:** Jing Chen, Hao-Ran Lu, Lei Zhang, Cheng-Hong Liao, Qian Han

**Affiliations:** 1 Key Laboratory of Tropical Biological Resources of Ministry of Education, Hainan University, Haikou, 570228 Hainan China; 2 Laboratory of Tropical Veterinary Medicine and Vector Biology, School of Life and Pharmaceutical Sciences, Hainan University, Haikou, 570228 Hainan China

**Keywords:** *DOPAL synthase*, *Aedes aegypti*, RNA interference, Development, Mortality

## Abstract

**Background:**

The cuticle is an indispensable structure that protects the mosquito against adverse environmental conditions and prevents pathogen entry. While most cuticles are hard and rigid, some parts of cuticle are soft and flexible to allow movement and blood-feeding. It has been reported that 3, 4-dihydroxyphenylacetaldehyde (DOPAL) synthase is associated with flexible cuticle formation in *Aedes aegypti*. However, the molecular function of *DOPAL synthase* in the ontogenesis of mosquito remains largely unknown. In this study, we characterized gene expression profiles of *DOPAL synthase* and investigated its functions in larvae and female adults of *Aedes agypti* by RNAi.

**Results:**

Our results suggest that the expression of *DOPAL synthase* is different during development and the transcriptional level reached its peak at the female white pupal stage, and *DOPAL synthase* was more highly expressed in the cuticle and midgut than other tissues in the adult. The development process from larva to pupa was slowed down strikingly by feeding the first-instar larvae with chitosan/*DOPAL synthase* dsRNA nanoparticles. A qRT-PCR analysis confirmed that the dsRNA-mediated transcription of the *DOPAL synthase* was reduced > 50% in fourth-instar larvae. Meanwhile, larval molt was abnormal during development. Transmission electron microscopy results indicated that the formation of endocuticle and exocuticle was blocked. In addition, we detected that the ds*DOPAL synthase* RNA caused significant mortality when injected into the female adult mosquitoes.

**Conclusions:**

Our findings demonstrate that *DOPAL synthase* plays a critical role in mosquito larval development and adult survival and suggest that *DOPAL synthase* could be a good candidate gene in RNAi intervention strategies in mosquito control.

**Electronic supplementary material:**

The online version of this article (10.1186/s13071-019-3568-7) contains supplementary material, which is available to authorized users.

## Background

The insect cuticle is a complex exoskeleton which covers all the exposed tissues and protects insects against adverse environmental conditions, forms a physical barrier against the intrusion of pathogens and maintains their movement [1–4]. It is also the main route of insecticide penetration, and the intake of insecticide can be reduced by thickening or changing the chemical composition of the cuticle [5]. The cuticle is mainly composed of chitin and cuticular proteins (CPs) [6–9]. Insects undergo molting several times throughout their lives. The new cuticle is soft and fragile, and it needs sclerotization, melanization and other physiological processes to play a protective role [8, 10, 11]. Cuticle sclerotization and melanization are important parts of the cuticle formation and hardening process. Tyrosine-mediated cuticle tanning is a vital step to achieve rapid formation of the protective cuticle. Tyrosine is first hydroxylated to 3,4-dihydroxyphenylalanine (DOPA), then DOPA is decarboxylated to dopamine by DOPA decarboxylase (DDC) [12]. Dopamine not only can be acetylated by arylalkylamine N-acetyltransferase (aaNAT) [13] or combined with N-β-alanine to form sclerotization precursors [14], but can also be oxidized by phenoloxidases to form melanization precursors [4, 10].

3,4-dihydroxyphenylacetaldehyde (DOPAL) synthase, which exists in most insect species according to sequence identity [11], can catalyze the conversion of L-DOPA to DOPAL which is involved in flexible cuticle formation. DOPAL synthase is a member of the insect DDC-like protein family. Although DOPAL synthase and DDC share high sequence similarity, they catalyze rather different chemical reactions. Structural and biochemical comparisons between DOPAL synthase and DDC in *Drosophila* identified that the key active site residue, Asn192, in insect DOPAL synthase can be used to distinguish all available insect DOPAL synthases from DDC sequences [15]. DOPAL has a high biological activity, which is directly linked to the CPs cross-linking through interaction of free amino acid groups on the CPs [11, 16, 17]. Early studies reported that DOPAL synthase-like protein, alpha methyldopa resistant (AMD-r), in *Drosophila* is resistant to the toxic compound, AMD. Homozygous mutations of *amd-r* resulted in embryo death during early development stages [18, 19]. Electron microscopic evidence revealed that loss of the gene function leads to cuticle defects in a region of *amd-r* expression [18]. These reports implied the role of the *amd-r* gene in flexible cuticle formation rather than the role of DDC in rigid cuticle melanization and sclerotization [20, 21]. However, the function of DOPAL synthase or AMD*-*r in the postembryonic development of insects is unknown.

Mosquitoes are the most dangerous disease vectors because they transmit deadly pathogens such as *Plasmodium* spp., chikungunya virus, yellow fever virus, dengue virus, Japanese encephalitis virus and West Nile virus [22]. Chemical insecticides have been widely used across the world for a long time. However, many classes of chemical insecticide have caused serious resistance in hundreds of mosquito species across sixty countries [23]. To address the problem of mosquito control, RNA interference (RNAi) has been increasingly used to conduct genetic studies in mosquito vectors and to study the evolution of insecticide resistance in mosquitoes [24–27].

RNAi is caused by double-stranded RNA (dsRNA), which degrades the homologous mRNA with high efficiency and specificity [28–30]. RNAi technology can be used to eliminate or knock down the specific expression of specific genes, so this technique has been widely used to explore gene function in physiological processes of different insect species. Improved administration and formulation of dsRNAs for insects coupled with the current progress in RNAi technology enables RNAi to be an alternative strategy for insect pest control [31–34]. Several methods of RNAi have been successfully used by researchers with their own advantages and limitations [24, 35, 36]. Microinjection of dsRNAs into adult insects is considered to be the most reliable delivery of the dsRNAs and has achieved high knockdown efficiency; however, this method is not applicable to larvae, especially the first- and second-instar larvae, since it demands a high level of skill from the operators [24, 36]. Oral delivery of dsRNAs can be facilitated by using a lipid-based transfection reagent in the larvae [37, 38]. However, it is susceptible to the intestinal environment. dsRNA is not stable, and the induction effect is inferior to the microinjection [36, 38]. Thus, dsRNA formulation of nanoparticles for larvae has attracted much attention. Chitosan encapsulates dsRNA to form nanoparticles which can make dsRNA release slowly and continuously, keep dsRNA to specific tissues outside the gut, and protect from digestion of intestinal nuclease and damage of pH change [39–42].

In the present study, we fed nanoparticles containing ds*DOPAL synthase* RNA to larvae and microinjected the dsRNA into adults to investigate the role of *DOPAL synthase* in cuticle information in *Aedes aegypti* mosquitoes. Our data demonstrated that *DOPAL synthase* plays a key role during the larvae development and adult survival in *Ae. aegypti*, and could be a good candidate gene in RNAi intervention strategies in mosquito control.

## Methods

### Mosquito rearing

*Aedes aegypti* mosquitoes (Rockefeller strain, provided by Beijing Institute of Microbiology and Epidemiology, Beijing, China) were used in the investigation. Mosquitoes were reared based on the previously reported procedure [43].

### Collection and dissection of mosquito tissues

To examine the temporal expression of *DOPAL synthase*, mosquito eggs, first- to fourth-instar larvae, white and black female pupae, white and black male pupae, female and male adults were collected for investigation. To examine the spatial expression of *DOPAL synthase*, different tissues of non-blood-fed female adults, including the head, thorax, cuticle and midgut were derived for the experiment. Tissues were dissected from around 30 mosquitoes, which were cold anesthetized on ice. The cuticles of male and blood-fed female adults were also collected. Around 10 individuals were used for each test and 3 tests were performed for each stage and tissue sample. A total of 30–50 first- and second instar-larvae were collected in each tube and 5–10 larvae of the other stages were collected in each tube.

### The expression profiles of *DOPAL synthase*

The collected samples were homogenized in Trizol reagent (Invitrogen, CA, USA) and total RNA was isolated according to the manufacturer’s instructions. Total RNA was treated with a PrimeScript™ RT reagent kit with gDNA Eraser (TaKaRa, Dalian, China) to remove DNA contamination from the samples and cDNA was synthesized for real-time PCR. Real-time PCR amplification and analysis were performed on a LightCycler® 96 (Thermo Fisher Scientific, Massachusetts, USA) using a Power SYBR Green Supermix kit (TaKaRa). Primers used for real-time PCR (forward: 5′-CAT TGG CAA ATT CAG CTC GG-3′ and reverse: 5′-CAT TGG CAA ATT CAG CTC GG-3′) were designed to amplify fragments of *DOPAL synthase* (XM_001661007). *gapdh* (glyceraldehyde 3 phosphate dehydrogenase) (XM_011494724) was used as an internal reference gene with primers (forward: 5′-TTG GAC TAC ACC GAA GAG GA-3′ and reverse: 5′-TGT CGT ACC AGG AGA TGA GC-3′). Amplifications were performed in a final volume of 10 μl containing 1 μl of cDNA, 1 μl of each primer, 4 μl of 2× SYBR Green Supermix, and 3 μl ddH_2_O. The thermal cycle conditions used for the real-time PCR were: 95 °C for 10 min, followed by 45 cycles of 95 °C for 10 s, 55 °C for 10 s and 72 °C for 10 s. Following the real-time PCR cycles, the specificity of the SYBR green PCR signal was confirmed by melting curve analysis or agarose gel electrophoresis. The mRNA expression was quantified using the comparative CT method (cross threshold, the PCR cycle number that crosses the signal threshold). The relative gene expression data were analyzed using the relative 2^−ΔΔct^ method [44].

### dsRNA preparation

Total RNA was extracted from *Ae. aegypti* adults with Trizol reagent (Invitrogen) and was then handled with a PrimeScript™ RT reagent kit with gDNA Eraser Kit (TaKaRa). A fragment of *DOPAL synthase* was PCR-amplified from the cDNA using the primers (forward: 5′-CCT GGA GGA AAT TGG TC-3′ and reverse: 5′-AGT AGG TGT CCT CTC CTT TCA-3′). The *β-glucuronidase* (*gus*) gene (KY848224), a bacterial gene specific to Escherichia *coli*, as a negative control, was amplified by PCR from the pBI121 plasmid using the primers (forward: 5′-GCG GCC GCC CCT TAC GCT GAA GAG ATG C-3′ and reverse: 5′-CTC GAG GGC ACA GCA CAT CAA AGA GA-3′) as reported [28]. Then, the PCR products were detected and purified using gel electrophoresis. The gene fragments were subcloned into the cloning vector PMD18-T (TaKaRa), and later excised from PMD18-T using *Sma*I and *Xba*I restriction enzymes, then ligated into plasmid pL4440, a vector possessing convergent T7 promoters. The recombinant plasmid were transformed into competent cells of HT115 (DE3) following a previously described method [28], which is an RNase-III-deficient E. coli strain, and the cells were grown in 2× LB medium (10 g/l of tryptone, 5 g/l of yeast extract, 10 g/l of NaCl) containing ampicillin and tetracycline at 37 °C for 12–14 h. The bacterial solution was diluted 100× with 2× LB medium and grown until OD600 = 0.5. IPTG was added in a final concentration of 0.6 mM to induce T7 polymerase activity. The expressed dsRNA was extracted and confirmed by electrophoresis on 1% agarose gel.

### Preparation of chitosan/dsRNA nanoparticles

Medium range chitosan (90–95% deacetylated) was purchased from BBI Life Sciences (Shanghai, China). Chitosan (1 mg/ml) was dissolved in sodium acetate buffer to obtain a 0.02% w/v working solution and the solution was kept at room temperature before use. A total of 32 μg of dsRNA was added to 1 μl of 50 mM sodium sulfate buffer, diethyl pyrocarbonate (DEPC) water was made up to 100 μl and then mixed with an equal volume of chitosan solution. The mixture was heated at 50 °C for 1 min, as vortexed for 30 s, and centrifuged at 13,000×*g* for 10 min to allow the formation of the nanoparticles. The percentage of dsRNA embedded in the nanoparticles was calculated using the starting amounts of dsRNA and amounts remaining in the supernatants by the absorbance measurement at 260 nm.

### Delivery of dsRNA to mosquito larvae

Chitosan/dsRNA nanoparticles were mixed with mosquito larval food consisting of agar (Biowest, Shanghai, China). The gel was cut into 6 equal slices using a clean razor blade or toothpick. One hundred age-synchronized first-instar larvae, 24 h after egg hatching, were placed into 10 Petri dishes in about 10 ml of deionized water. Larvae were fed one slice per Petri dish each day. The procedure was repeated daily until the larvae were raised to the fourth instar. The transcript levels were tested and other phenotypic changes were observed in different larval stages. The negative control gel with ds*gus* was prepared in the same way. The black control was directly fed with food without using nanoparticles.

### Quantitative RT-PCR to measure gene knockdown

Quantitative RT-PCR (qRT-PCR) assays were performed with three biological replicates on at least 10 pooled control *vs* experimental larvae as described. Larvae from each treatment were collected and pooled together each instar after dsRNA feedings. RNA extractions and cDNA syntheses were performed as mentioned above. The cDNA from each replicate treatment was then used to assess the extent of RNAi by measuring levels of gene expression using qRT-PCR. Reactions were performed in triplicate using the primers listed as above. The *gapdh* gene was used as an internal reference to compare levels of RNAi. Melt curve analyses were also performed and confirmed that only a single product was amplified with each primer pair in every sample. Analysis of gene expression was performed using the 2^−ΔΔCT^ method, comparing expression in specific dsRNA treated samples to *gus*-dsRNA treated samples.

### Transmission electron microscope (TEM) analysis

The cuticle of *Ae. aegypti* in the abdomen were dissected from the native larvae of the first to fourth instar (sampling immediately after molting) (*n* = 10), and RNAi-treated fourth-instar larvae (*n* = 10). Normal cuticles of the wild larvae groups in the same instar were used as a blank control. Specimens were fixed in 2.5% glutaraldehyde (Kemiou, Tianjin, China) in phosphate buffer (0.1 M, pH 7.0) overnight, and samples were washed 3 times for 5 min with phosphate buffer. Specimens were further fixed with 1% osmium tetroxide buffer in phosphate buffer (0.1 M, pH 7.0) at 4 °C for 1–3 h. After fixation, specimens were washed 3 times with phosphate buffer (0.1 M, pH 7.0) for 5 min. The samples were gradually dehydrated with 30, 50, 70, 90, 95 and 100% ethanol for 5 to 10 min at each step; the 100% ethanol step was repeated 2 to 3 times to ensure complete dehydration. After dehydration, the samples were embedded in epoxy resin (Sigma-Aldrich, St. Louis, USA). The specimens were sliced with an ultramicrotome (Leica UC6; Leica, Vienna, Austria). The sections were stained with uranyl acetate (Syntechem, Changzhou, China) and lead citrate (Acros, Shanghai, China) and observed using an electron microscope (JEM1200EX; Jeol, Tokyo, Japan).

### Injection of dsRNA into adult mosquitoes

*Aedes aegypti* adults were collected 3–5 d after hatching and in 4–7 h after they had fed on sugar water. The number of mosquitoes were controlled at around 300 at a time, and put into a refrigerator at − 20 °C for 4 min. The frozen mosquitoes were put on ice and purified dsRNA was injected into the side of their chests using an oil pressure manual microinjection device (Eppendorf AG, Hamburg, Germany). Meanwhile, three groups were injected with the dsRNA of *gus*, DEPC water and native treatment, respectively, as controls. Each time, 100 female and 100 male were injected and all experiments were repeated for three times. Each sample was injected with 3 μl into the thorax.

### Statistical analyses

A one-way ANOVA test was performed to evaluate the significance of the difference between the groups. The threshold level of significance was *P < *0.05.

## Results

### Spatiotemporal expression profiles of the *DOPAL synthase* gene

**Fig. 1:**
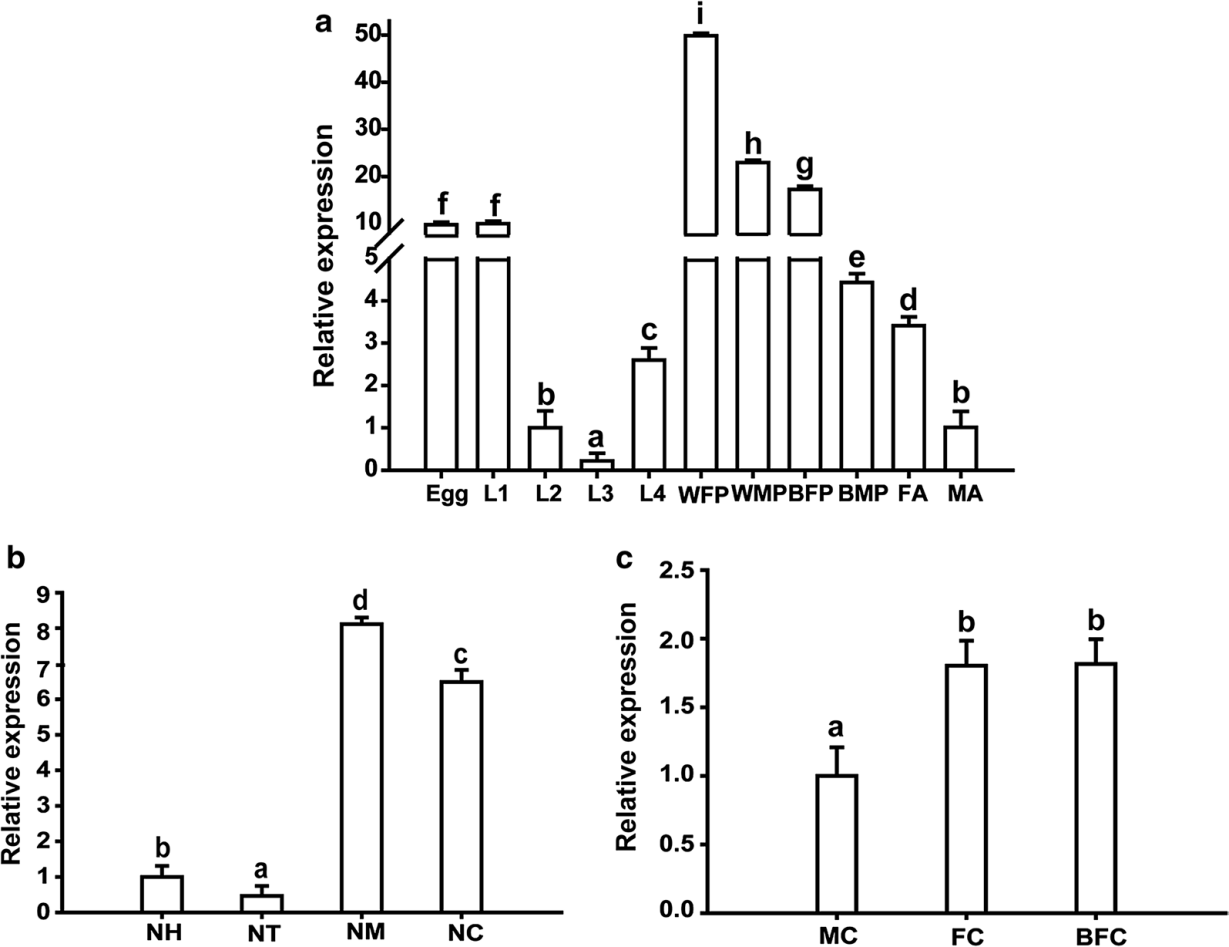
The expression profiles of *DOPAL synthase* gene with *gapdh* as the reference gene. Bars represent the standard deviation. **a** Temporal expression of *DOPAL synthase* in different developmental stages. **b** Spatial expression of *DOPAL synthase* in different tissues of *Ae. aegypti.* **c** Sex-different expression of *DOPAL synthase* in the cuticle of female and male adults. *Abbreviations*: L1, first-instar larvae; L2, second-instar larvae; L3, third-instar larvae; L4, fourth-instar larvae; WFP, white female pupae; WMP, white male pupae; BFP, black female pupae; BMP, black male pupae; FA, female adults; MA, male adults; NH, head; NT, thorax; NM, midgut; NC, cuticle; MC, cuticle of male adults; FC, cuticle of female adults; BFC, cuticle of blood-fed female adults

Data showed that the *DOPAL synthase* gene is differently expressed throughout the life of *Ae. aegypti*. The temporal expression profile was determined in the larval, pupal, male and female adult stages. Our test showed that the *gapdh* gene has a similar expression trend with the *rps17* gene (40S ribosomal protein s17) (Additional file 1: Figure S1), which is a reference gene, during qPCR for late embryos, all four larval-instars and pupae samples in *Aedes albopictus* [45]. In addition, *gapdh* in this study as reference gene was deemed sufficient, as the PCR efficiencies of the primer sets were greater than 95%, calculated using the method of Pfaffl [46]. The expression levels of *DOPAL synthase* were higher in the first-instar than in the other three instars; expression then peaked in the white female pupae and reduced in black pupae and adults. In addition, female adults had significantly higher expression of *DOPAL synthase* than male adults (Fig. 1a).

qRT-PCR showed that *DOPAL synthase* expression in different tissues of *Ae. aegypti* adults was also different. It was highly expressed in the cuticle and midgut of non-blood-fed mosquitoes. The expressions in the head and thorax were relatively low (Fig. 1b). The expression of *DOPAL synthase* in the male mosquito cuticle was the lowest, and there was no difference between non-blood-fed and blood-fed females (Fig. 1c).

### RNAi and phenotypes in mosquito larvae

**Fig. 2:**
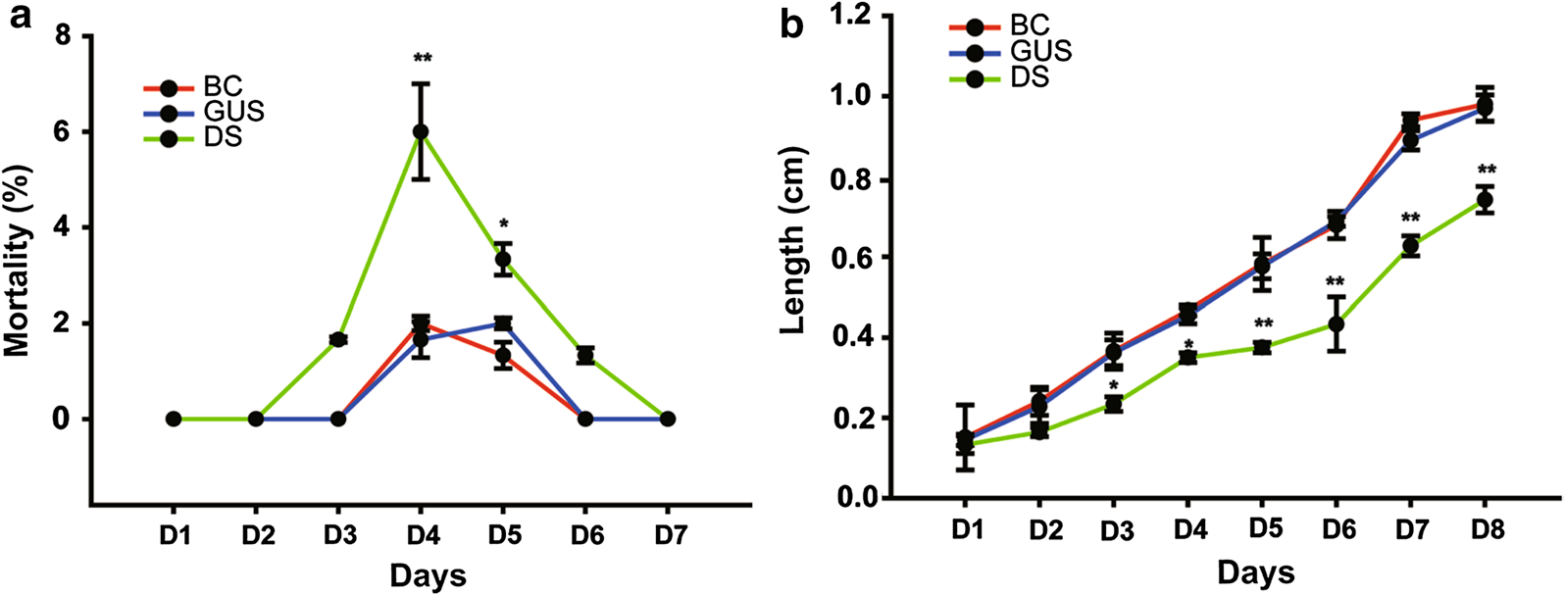
Treatment of mosquito larvae with *DOPAL synthase* dsRNA increased mortality of larvae and slowed in larval development. Bars represent the standard deviation. **a** The mortality of larvae after treatment with *DOPAL synthase* dsRNA. **b** The length of larvae after treatments with *DOPAL synthase* dsRNA. *P < 0.05, **P < 0.01. *Abbreviations*: DS, treatment of larvae with ds*DOPAL synthase*; GUS, treatment of larvae with ds*gus* RNAs; BC, black control

**Fig. 3:**
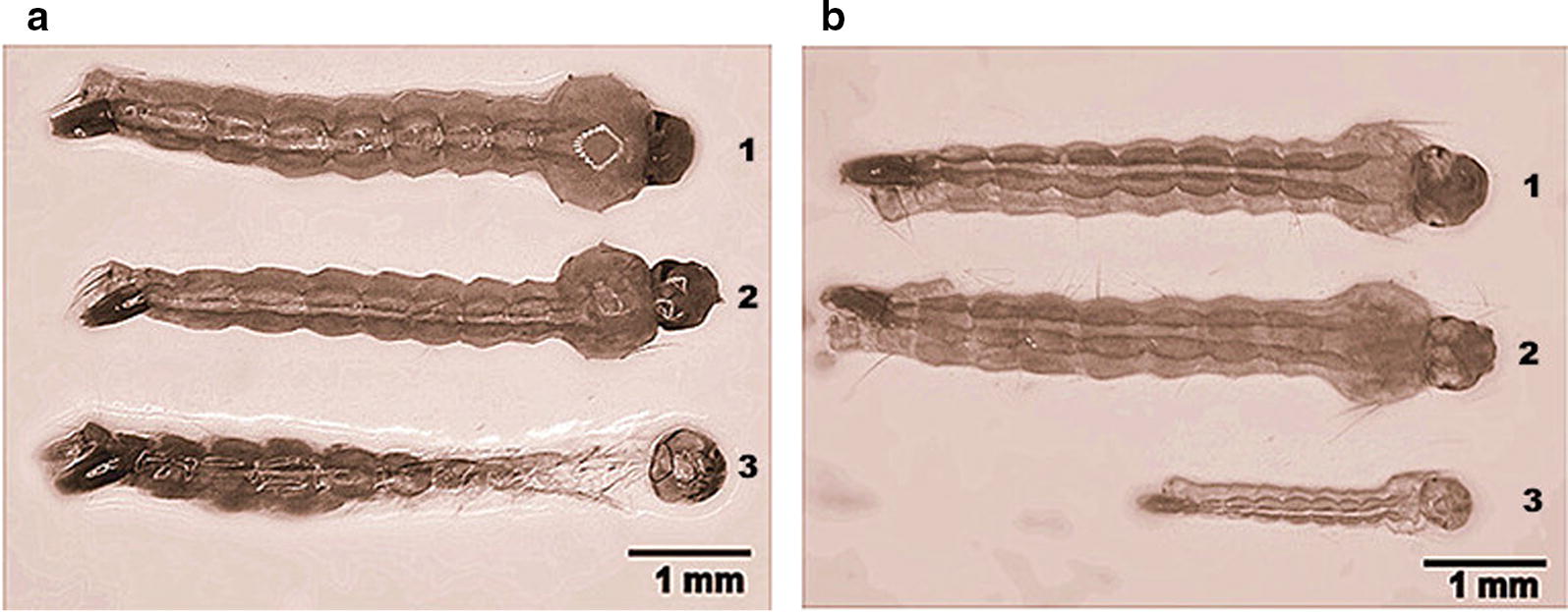
Morphological changes of the larvae fed on nanoparticles of the dsRNAs. **a** Compared with the third-instar larvae of the blank control (1) and those fed on ds*gus* (2), the dead larva which had fed on *DOPAL synthase* dsRNA were unable to detach from the exuvia (3). **b** Compared with the fourth-instar larva of the blank control (1) and those fed on ds*gus* (2), the larva which had fed on *DOPAL synthase* dsRNAs showed a much smaller size (3)

In larvae fed on *DOPAL synthase* dsRNA, the gene silencing led to high mortality compared with the negative controls. The mortality rate of larvae fed on *DOPAL synthase* dsRNA was nearly three times higher than that of the two control groups (ANOVA, *F*_(3,9)_ = 9.81, *P* = 0.013) (Fig. 2a). Most larvae in the experimental group grew slowly compared with the control groups (Fig. 2b). The length of *DOPAL synthase* dsRNA-treated larvae was significantly less than that of the control groups from the third day after gene silencing (ANOVA, *F*_(3,9)_ = 326.38, *P < *0.0001) and the disparity was about 1–2 times (Fig. 2b). Many larvae died from *DOPAL synthase* dsRNA treatment, with incomplete molting as observed under a microscope (Fig. 3a). At the same time, the silence of *DOPAL synthase* arrested the emergence of larvae into pupae compared with ds*gus* and black control (Table 1). As shown in Table 1, the larvae stopped growing and development, 10% of the larvae in the experimental group stopped at the second-instar compared with the two control groups (Fig. 3b).

**Table 1:** Statistics on the number of abnormal larvae

	1^a^	2^a^	3^a^	Mean ± SD
DS	13	9	8	10 ± 2.65^b^
GUS	3	0	2	1.67 ± 1.53
BC	1	1	2	1.33 ± 0.577

^a^1, 2, 3, three biological replicates

^b^The number of abnormal larvae in *DOPAL synthase*-dsRNA-treated group was much higher compared with *gus*-dsRNA treated larvae and blank control (one-way ANOVA, *P < *0.01)

*Abbreviations*: DS, treatment of larvae with ds*DOPAL* synthase; GUS, treatment of larvae with ds*gus* RNAs; BC, blank control

### The efficiency of RNAi in larvae

**Fig. 4:**
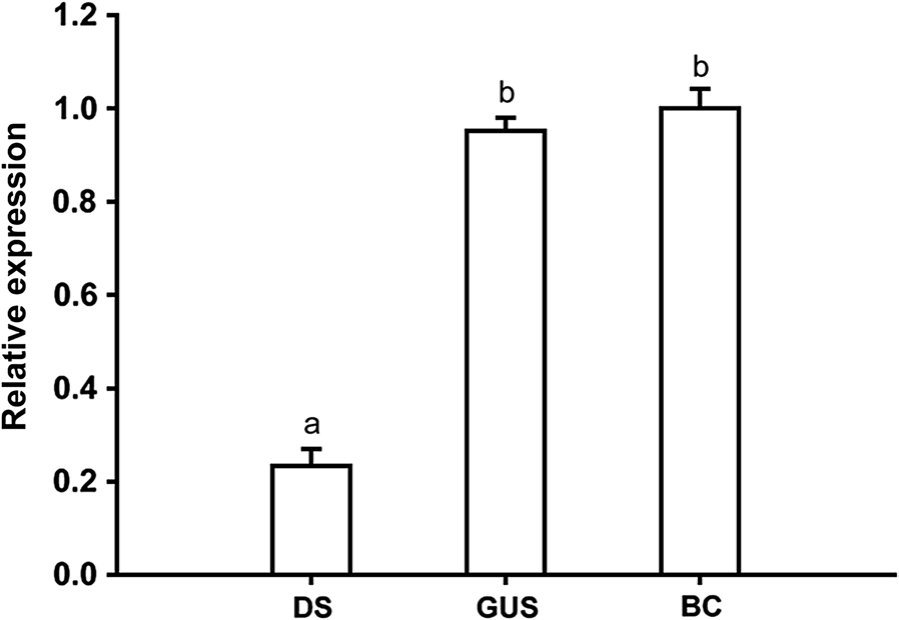
Quantitation of the expressions of *DOPAL synthase* after RNAi of larvae. Relative transcript levels of *DOPAL synthase* in the larvae after being continuously fed on *DOPAL synthase* dsRNA or *gus* dsRNA (ds*gus* as a control). The data are presented as means ± SD of three replicate samples. Bars represent the standard deviation. Different letters indicate statistically significant differences. *Abbreviations*: DS, treatment of larvae with ds*DOPAL synthase*; GUS, treatment of larvae with ds*gus* RNAs; BC, black control

Our results showed that feeding the mosquito larvae with *DOPAL synthase* dsRNA nanoparticles effectively triggered RNAi in the larvae. The expression shown in Table 2 demonstrates that the transcript levels of *DOPAL synthase* are repressed about 80% by *DOPAL synthase* dsRNAs and there is no significant difference between the ds*gus* fed group and black control (Fig. 4).

**Table 2:** Quantitation of the expressions of *DOPAL synthase*^a^ after RNAi of larvae

	1^b^	2^b^	3^b^	Mean ± SD
DS	0.23	0.24	0.31	0.26 ± 0.043^c^
GUS	0.90	0.95	0.97	0.95 ± 0.036
BC	1	1	1	1 ± 0.047

^a^Level of mRNAs relative to blank control transcripts with 2^-△△ct^ method

^b^1, 2, 3, three biological replicates

^c^The *DOPAL synthase* gene expression in *DOPAL synthase*-dsRNA treated group was higher compared with *gus*-dsRNA treated larvae and blank control (one-way ANOVA, *P < *0.01)

*Abbreviations*: DS, treatment of larvae with dsDOPAL synthase; GUS, treatment of larvae with dsgus RNAs; BC, blank control

### Transmission electron microscope (TEM) analysis

**Fig. 5:**
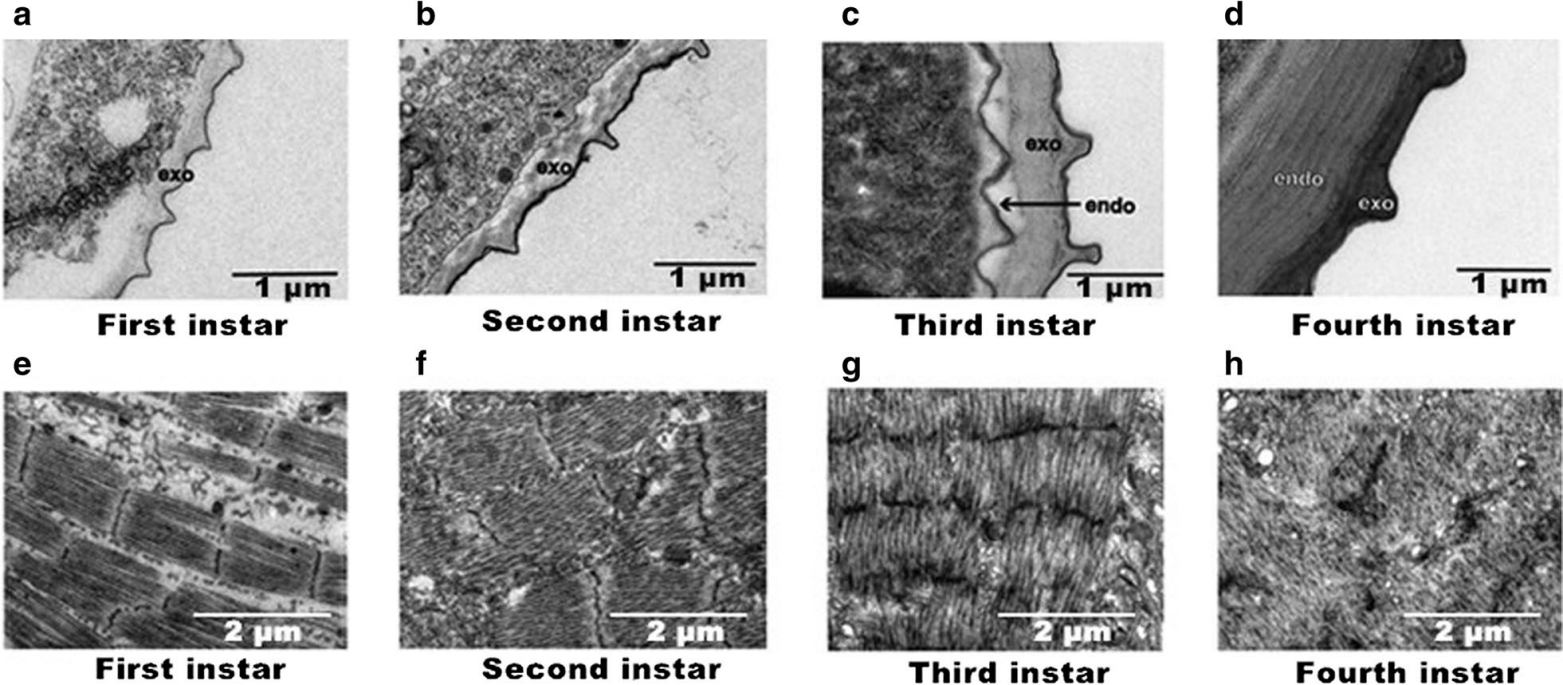
Morphology of vertical and horizontal sections of larval abdominal integument of different larval stages by TEM. **a**–**d** Vertical sections of integument from the first- to the fourth-instar larvae, respectively. **e**–**h** Horizontal sections of integument of the first- to fourth-instar larvae, respectively. *Abbreviations*: exo, exocuticle; endo, endocuticle

**Fig. 6:**
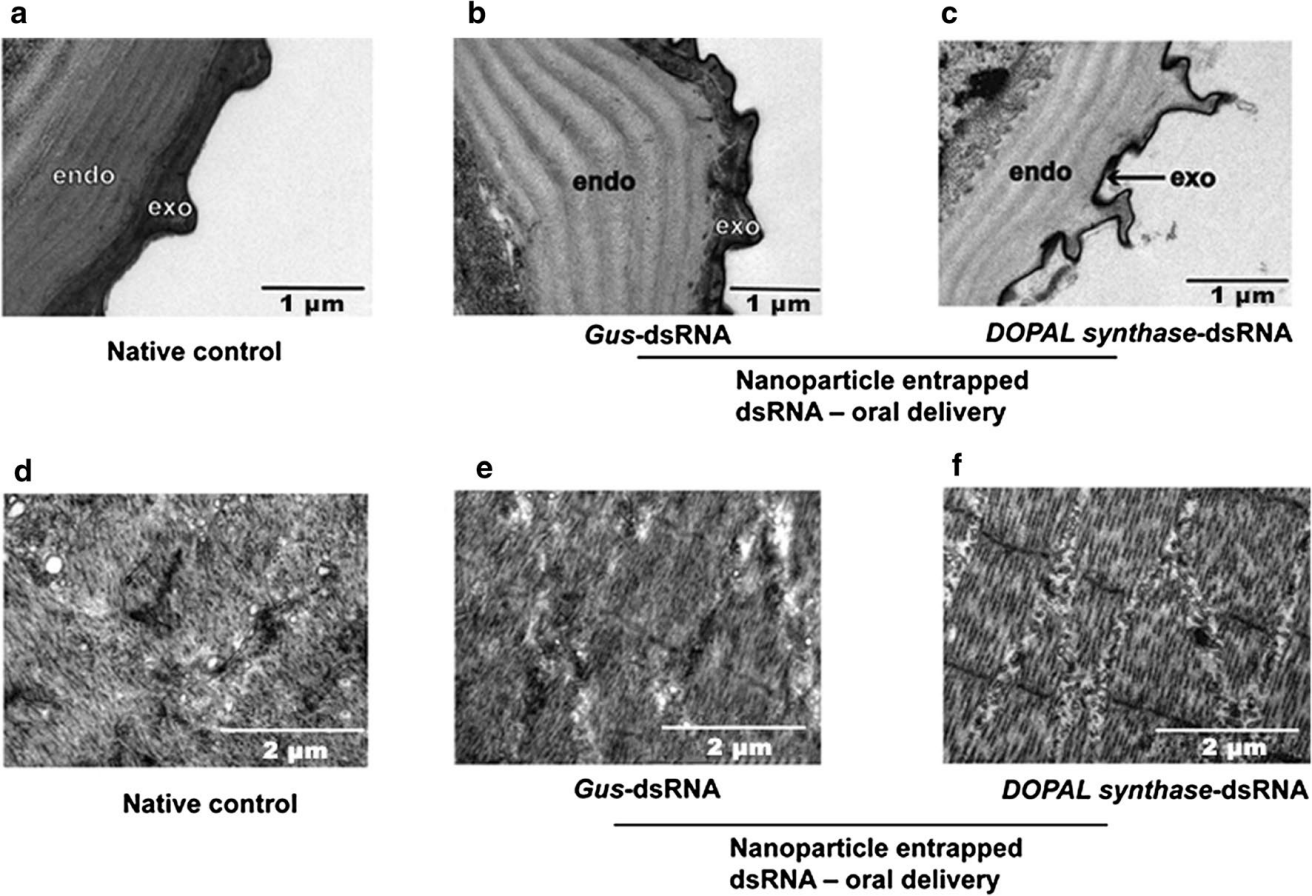
Morphology of vertical and horizontal sections of larval abdominal integument after treatment with *DOPAL synthase* dsRNA. Larvae treated with ds*gus* and without treatment were used as controls. **a**, **d** Vertical and horizontal sections of integuments of the control without treatment, respectively. **b, e** Vertical and horizontal sections of integuments of larvae which were treated with ds*gus*, respectively. **c**, **f** Vertical and horizontal sections of integument of larvae which were treated with *DOPAL synthase*-dsRNA, respectively

TEM detection of the larval abdomen collected from first-instar to fourth-instar larvae showed the RNAi effect on the development of cuticle. The insect cuticle is composed of the epicuticle and procuticle, and the latter is made up of the exocuticle and endocuticle [4]. The vertical section results showed that the endocuticle is deposited at the third-instar stage and becomes thicker at the fourth instar stage (Fig. 5a–d). In addition, the horizontal section results indicated that the surface texture of the larvae gradually becomes irregular and chaotic from the first- to the fourth-instar larvae (Fig. 5e–h). In order to understand the role of *DOPAL synthase* on the development of the larval cuticle, microstructural analysis of the abdomen integuments of the fourth-instar larvae was performed by TEM (Fig. 6). The integuments of larvae untreated (Fig. 6a) and treated with ds*gus* (Fig. 6b) were normal. However, the mosquitoes treated with *DOPAL synthase* dsRNA showed that the cuticle microstructure had only four endocuticle lamellae without the exocuticular deposited (Fig. 6c) compared with the mosquitoes in ds*gus*-treated (Fig. 6b) and untreated (Fig. 6a) groups. The interference of *DOPAL synthase* can also result in an abnormal surface texture of larval integument at the second-instar larvae, as illustrated in Fig. 6f, compared with the larvae in the ds*gus*-treated (Fig. 6e) and untreated (Fig. 6d) groups. These results reveal that *Ae. aegypti DOPAL synthase* is indispensable for the normal formation of the endocuticle and the larval development.

### RNAi leads to high mortality of adults

**Fig. 7:**
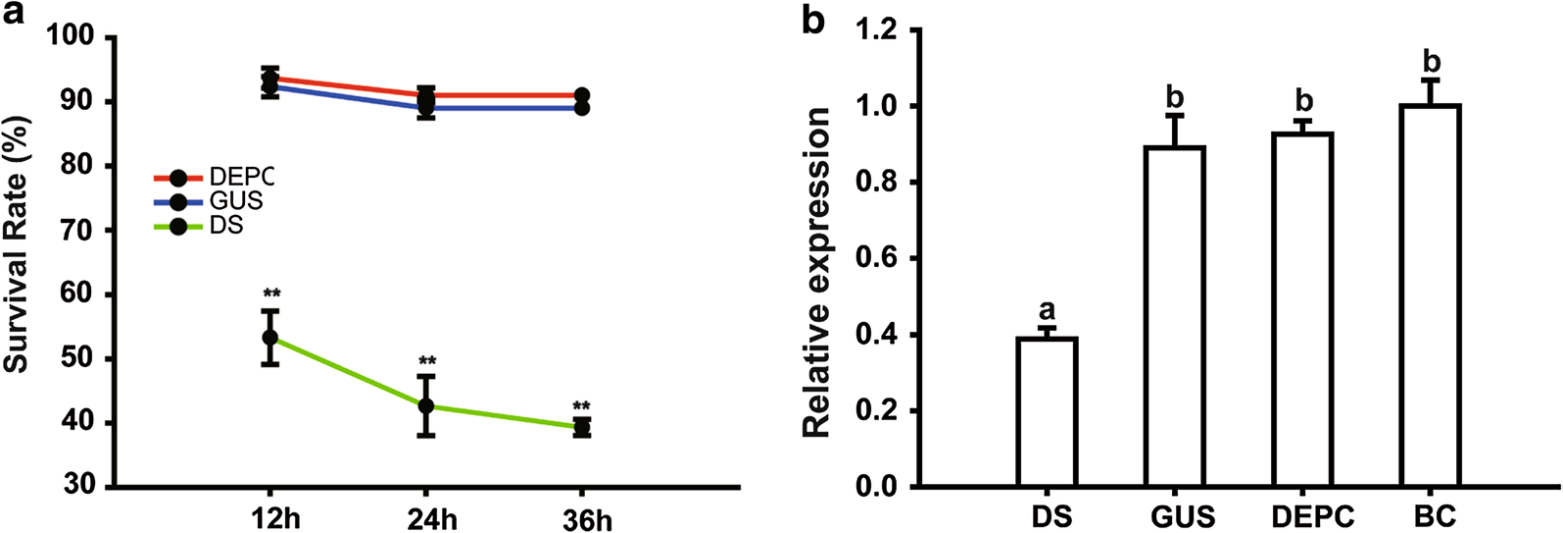
The survival curve and interference of *DOPAL synthase* transcription after injection of *DOPAL synthase* dsRNA. Bars represent the standard deviation. Different letters indicate statistically significant differences, **P < 0.01. **a** The survival rate of mosquitoes at 12, 24 and 36 h after injection. **b** Knockdown of *DOPAL synthase* transcription by injection. *Abbreviations*: DS, treatment of adult mosquitoes with *DOPAL synthase*-dsRNA; GUS, treatment of adult mosquitoes with *gus*-dsRNA; DEPC, treatment of adult mosquitoes with DEPC water; BC, black control

Most male mosquitoes died after they were injected with water or dsRNA. Therefore, we performed injection on the female mosquitoes to elucidate the function of *DOPAL synthase*. As shown in Fig. 7, the results of the dsRNA injection showed that *DOPAL synthase* RNAi can cause mortality in female adults, and the mortality rate was significantly higher than that of the control groups (Fig. 7a). At 12 h post-inoculation, the mortality was 46.6 ± 4.2% in dsRNA-inoculated mosquitoes, but mortality was 7.66 ± 1.6% and 6.3 ± 1.5% in the *gus*-dsRNA-inoculated and DEPC water-inoculated mosquitoes, respectively. At 24 h post-inoculation, the cumulative mortality of *DOPAL synthase* dsRNA-inoculated mosquitoes progressively increased to 57.2 ± 4.6%; *gus*-dsRNA -inoculated mosquitoes and DEPC water-inoculated were only 10.0 ± 2.7% and 8.9 ± 1.2%, respectively. In summary, the mortality of adult mosquitoes treated with *DOPAL synthase*-dsRNA was over 50%, while those in the control groups were only about 10% each (Fig. 7a).

At 12 h post-injection, RNA was extracted from adult mosquitoes for testing *DOPAL synthase* expression using qRT-PCR. As shown in Fig. 7b, the levels of *DOPAL synthase* transcript in *DOPAL synthase* dsRNA-inoculated mosquitoes were significantly reduced as compared with those in ds*gus*-inoculated, DEPC water-inoculated and non-treated mosquitoes. The results indicated > 60% mRNA knockdown with *DOPAL synthase* dsRNA.

## Discussion

The insect cuticle has the function of protection and defense against pathogen infection and environmental stress factors, and plays an important role in the life of insects [3, 8]. The insect cuticle undergoes sclerotization, melanization and other physiological processes to play a protective role [8, 10, 11]. DOPAL synthase can catalyze the reaction from L-dopa to DOPAL, which can interact with free amino acids of CPs and directly participate in surface protein cross-linking [15–17]. However, there is no known biological effect of DOPAL in the postembryonic development of mosquitoes. In this study, we demonstrated the function of *DOPAL synthase* in larvae and adults of *Ae. aegypti* mosquitoes by RNAi and in order to better understand the roles of *DOPAL synthase* during post-embryonic development.

In the present study, it was found that the expression of *DOPAL synthase* was the highest in white female pupae and mainly distributed in the cuticle (Fig. 5). The results suggest that *DOPAL synthase* may be involved in soft and flexible cuticle formation in the developmental stages from pupa to female adult. The exocuticle is the main new cuticle that is deposited in each instar post-ecdysis, and the endocuticle is accumulated from the third-instar larvae, then becomes thicker with each layer deposition in the fourth-instar larvae. During the early development of *Drosophila melanogaster*, homozygous mutations of *DOPAL synthase*-like gene, *amd-r*, resulted in embryo death [18, 19]. By reducing *DOPAL synthase* expression through RNAi, we found that the mortality rate of larvae was increased, and some larvae died due to failure of molting. It is worth noticing that there are obvious flaws in the abdominal cuticle of the arrested developmental larvae, as seen with transmission electron microscopy. The procuticle, composed of the exocuticle and the endocuticle, was thinner in the DOPAL synthase RNAi-injected group than in the controls. In addition, *DOPAL synthase* RNAi can also lead to changes in integument structure, whereby its surface texture becomes more regular and ordered, which may lead to poor flexibility of the integument. Based our results, it can be proved that *DOPAL synthase* is associated with the formation of cuticle structure, particularly of the endocuticle, and that larval development could be stopped at any of the larval stages if *DOPAL synthase* is totally knocked out in mosquitoes.

To identify if the dsRNA delivery was by feeding or by absorption through the larval cuticle, the dsRNA was mixed with a visible dye to identify those individuals that absorb the solution to assess the extent of ingestion of the dsRNA solution. The results showed that the dye, bromophenol blue, was mostly found in the alimentary tract, and little found in other tissues (Additional file 2: Figure S2), suggesting that the dsRNA delivery was mainly through feeding by larvae.

We know that juvenile hormones are the major hormonal regulator during the development of insects, which also can regulate cuticle formation [47–50]. Whether DOPAL synthase, juvenile hormone and ecdysone have any interactions *via* metabolism pathway cross-talk or any other interactions in regulating cuticle formation should be further studied. Moreover, RNAi of *DOPAL synthase* also leads to the massive death of female adult mosquitoes treated with *DOPAL synthase* dsRNA. The mortality rate is up to 50% compared with the controls. The levels of *DOPAL synthase* transcript in the dsRNA-inoculated mosquitoes were also significantly reduced 12 h after inoculation (Fig. 7a). Microinjection of the dsRNA into adult insects is considered to be the most reliable dsRNA administration and has achieved high knockout efficiency [51]. In this study, such rapid interference of *DOPAL synthase* is surprising. Although the mechanism underlying the mortality in adult resulted by the *DOPAL synthase* dsRNA treatment remains to be determined, we speculate that *DOPAL synthase* plays a more critical role in adults than in larval stages, which may explain the fact that interference of the gene expression has more significant effects on adults than on larvae. The previous hypothesis proposed that the gene may be associated with the feeding ability of adults, particularly blood-feeding of female adults by forming more a flexible cuticle [17, 52], which allows the mosquito to enlarge its body to have a large enough meal. Adults have less chance to access food than larvae; they need to eat enough food once they have a host or a food source.

Our findings demonstrate that RNAi of *DOPAL synthase* gene expression results in larval development stagnation, abnormality of cuticle structure, adult death, and other molting behavioral alterations. It may, therefore, be inferred that *DOPAL synthase* is an essential gene for mosquito development and survival and thus implies its worth as a target for mosquito control.

## Conclusions

This study provides evidence for the functions of *DOPAL synthase* during larvae development and adult survival in mosquitoes. Interference of *DOPAL synthase* can affect the molting and normal development of larvae, and leads to the death of adult mosquitoes. Our results suggest that *DOPAL synthase* is a potential target for mosquito control using RNAi technology.

## Additional files

**Additional file 1: Figure S1.** The expression profiles of *DOPAL synthase* gene with *rpf17* as the reference gene. Bars represent the standard deviation. Different letters indicate statistically significant differences (*P* < 0.05). **a** Temporal expression of *DOPAL synthase* in different developmental stages. **b** Spatial expression of *DOPAL synthase* in different tissues of *Ae. aegypti.* **c** Sex-different expression of *DOPAL synthase* in the cuticle of female and male adults. *Abbreviations*: L1, first-instar larvae; L2, second-instar larvae; L3, third-instar larvae; L4, fourth-instar larvae; WFP, white female pupae; WMP, white male pupae; BFP, black female pupae; BMP, black male pupae; FA, female adults; MA, male adults; NH, head; NT, thorax; NM, midgut; NC, cuticle; MC, cuticle of male adults; FC, cuticle of female adults; BFC, cuticle of blood-fed female adults.

**Additional file 2: Figure S2.** Double-stranded RNA mediated RNA interference with a dye by soaking. **a** The fourth-instar larvae after being soaked in dsRNAs with bromophenol blue, and without the dye and dsRNA (blank control). 1, blank control; 2, the dye and ds*DOPAL synthase* treated; 3, the dye and ds*gus* treated. **b** The dye distribution in alimentary tract and other tissues. 1, the midgut of ds*DOPAL synthase* and the dye treated larva; 2, the other extraintestinal tissues of ds*DOPAL synthase* and the dye treated larva; 3, the midgut of ds*gus* and the dye treated larva; 4, the other extraintestinal tissues of ds*gus* treated larva and the dye; 5, the midgut of the blank control (without being given dsRNA and dye) larva; 6, the other extraintestinal tissues of the blank control larva.

## Data Availability

The data supporting the findings of this article are included within the article and its additional files.

## Abbreviations

DOPAL: 3,4-dihydroxyphenylacetaldehyde
DDC: DOPA decarboxylase
RT-PCR: real-time polymerase chain reaction
RNAi: RNA interference
TEM: transmission electron microscope
