# Supplementary figures and images for: RNA interference-mediated knockdown of 3, 4-dihydroxyphenylacetaldehyde synthase affects larval development and adult survival in the mosquito Aedes aegypti
Source: Parasit Vectors. 2019 Jun 24;12:311. doi: 10.1186/s13071-019-3568-7 (PMC6591897; doi:10.1186/s13071-019-3568-7)

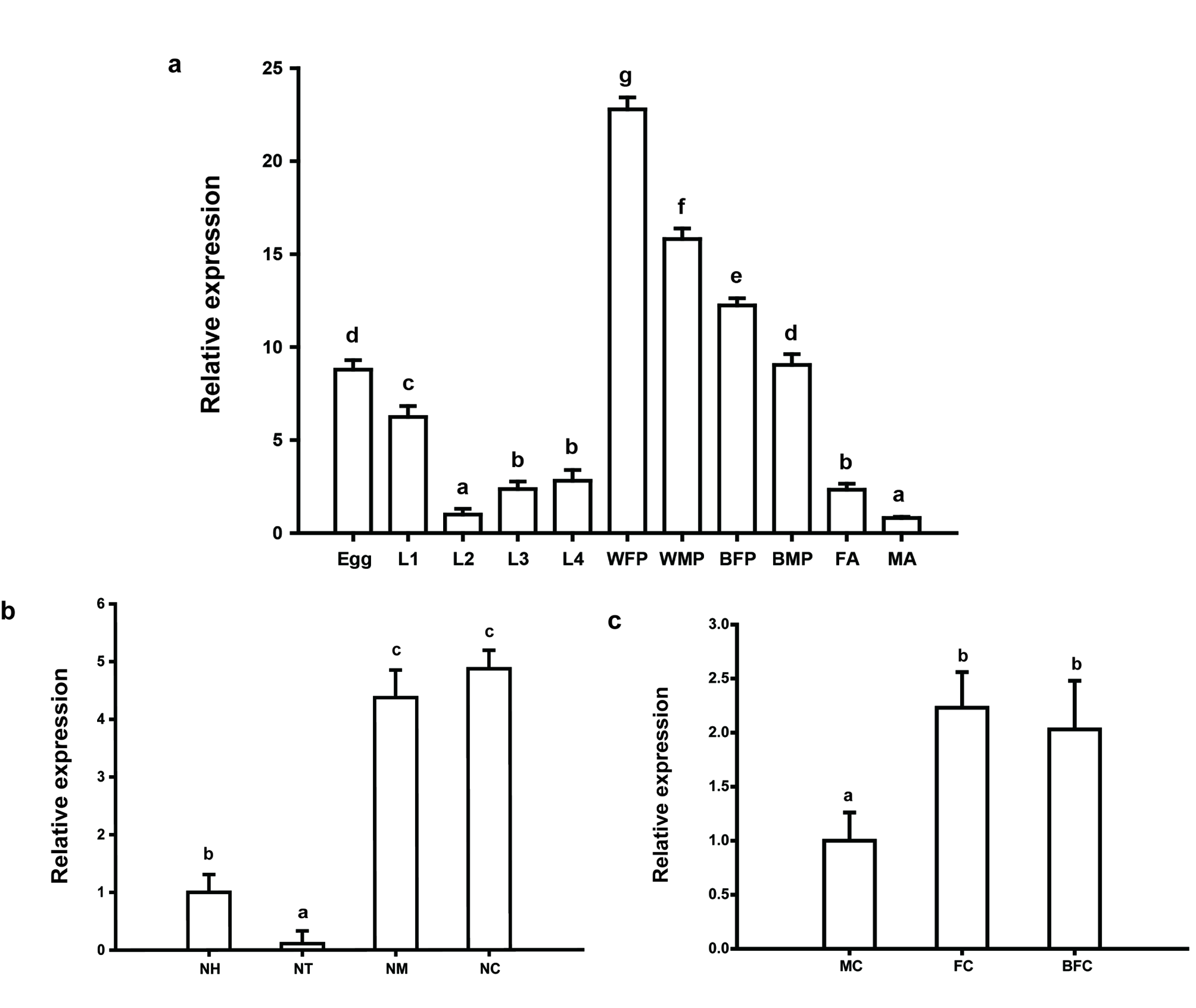

Supplement: Supplementary file 1 — Additional file 1: Figure S1. The expression profiles of DOPAL synthase gene with rpf17 as the reference gene. Bars represent the standard deviation. Different letters indicate statistically significant differences (P < 0.05). a Temporal expression of DOPAL synthase in different developmental stages. b Spatial expression of DOPAL synthase in different tissues of Ae. aegypti. c Sex-different expression of DOPAL synthase in the cuticle of female and male adults. Abbreviations: L1, first-instar larvae; L2, second-instar larvae; L3, third-instar larvae; L4, fourth-instar larvae; WFP, white female pupae; WMP, white male pupae; BFP, black female pupae; BMP, black male pupae; FA, female adults; MA, male adults; NH, head; NT, thorax; NM, midgut; NC, cuticle; MC, cuticle of male adults; FC, cuticle of female adults; BFC, cuticle of blood-fed female adults. [file 13071_2019_3568_MOESM1_ESM.tif]

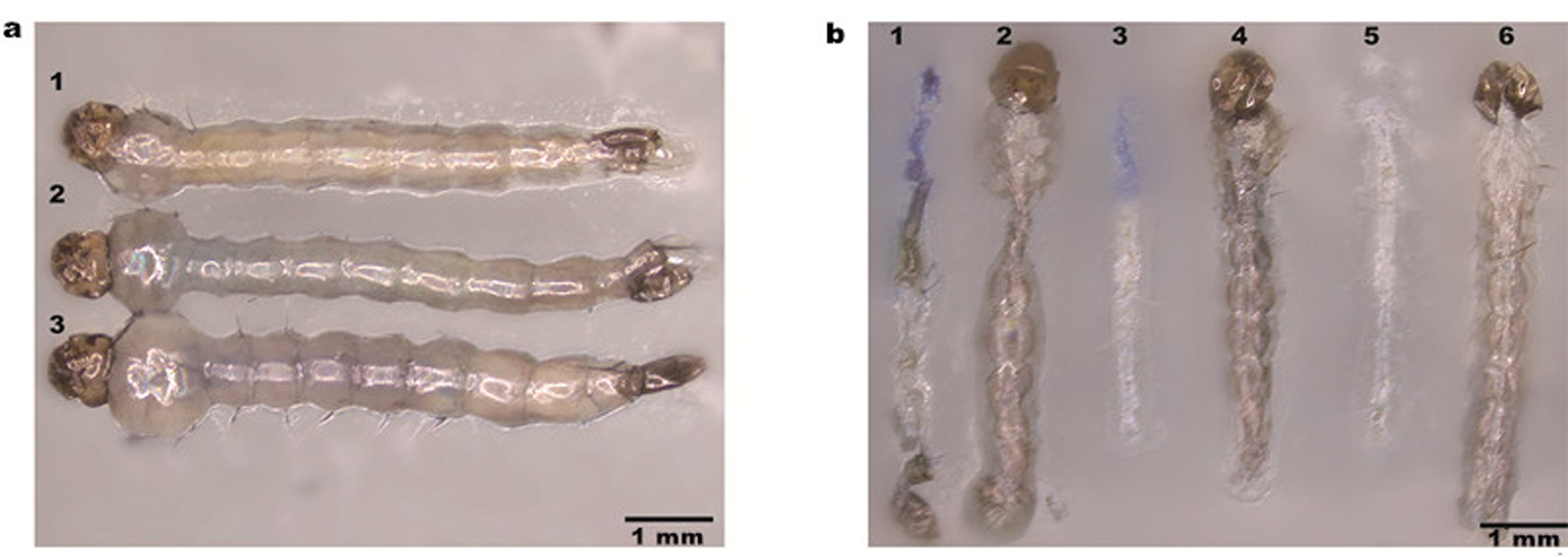

Supplement: Supplementary file 2 — Additional file 2: Figure S2. Double-stranded RNA mediated RNA interference with a dye by soaking. a The fourth-instar larvae after being soaked in dsRNAs with bromophenol blue, and without the dye and dsRNA (blank control). 1, blank control; 2, the dye and dsDOPAL synthase treated; 3, the dye and dsgus treated. b The dye distribution in alimentary tract and other tissues. 1, the midgut of dsDOPAL synthase and the dye treated larva; 2, the other extraintestinal tissues of dsDOPAL synthase and the dye treated larva; 3, the midgut of dsgus and the dye treated larva; 4, the other extraintestinal tissues of dsgus treated larva and the dye; 5, the midgut of the blank control (without being given dsRNA and dye) larva; 6, the other extraintestinal tissues of the blank control larva. [file 13071_2019_3568_MOESM2_ESM.tif]
